# Excision of Basal Cell Carcinoma with Radio Frequency Ablation

**DOI:** 10.4103/0974-2077.41157

**Published:** 2008-01

**Authors:** Sukhdeo Patidar

**Affiliations:** *Baroda Skin Clinic, Saraswati Complex, Nanjalpur, Vadodara, Gujarat, India*

**Keywords:** Basal cell carcinoma, Radiofrequency, Ablation

## Abstract

Basal cell carcinoma is usually treated by excision, or by ablative methods such as cryosurgery and laser. We describe treament of basal cell carcinoma by radiofrequency device.

## INTRODUCTION

Basal cell carcinoma (BCC) is commonest in whites, but not rare in pigmented population.[1] It comprises 65% of all skin malignancies and 95% patients are above 40 years age. Tumour is common on eyelid, inner canthus and behind the ear, uncommon on limbs and back, rare on vermilion of lips, palms and soles. In distribution of lesion, the density of pilosebaceous follicles is an important determining factor. BCC are more common in males. Outdoor occupations with increase sun exposure and sunburns, ionizing radiation are also important factors. BCC can also arise in burns scars, nevus sebaceous and melanocytic nevus.

## CASE REPORT

**Figure 1:**
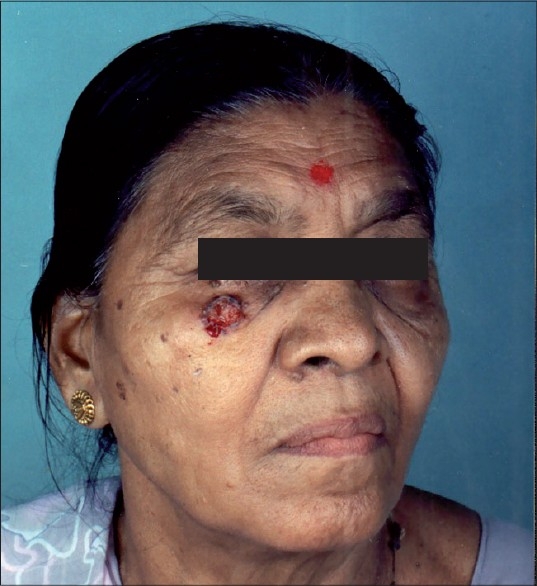
Non-tender, indurated ulcer with raised margins over right infraorbital region

**Figure 2:**
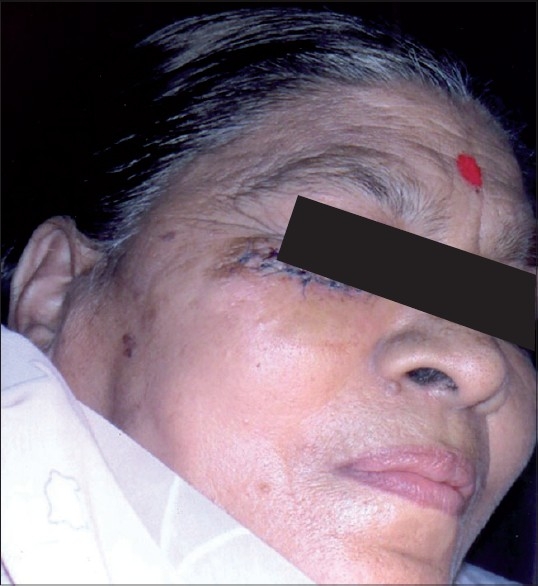
Primary closure with Prolene™ after radiofrequency excision

A 48-year-old woman developed 1.5 × 1.5 cm^2^ non-healing ulcer, 1 cm below right eyelid of 8 months duration [Figure 1]. It had increased to this size from a small papule. On examination, the ulcer was round, non-tender, and easily bled on slight touch. Edges were rolled up with crusted base. Biopsy by 3.5-mm punch was done and it confirmed the diagnosis of BCC. Under field block and local infiltration ulcer was excised with radio frequency (RF) cutting mode as per the standard procedure.[2] Five millimetres normal skin margin was also included in excision and sent for histopathology. The incision was elliptical which was done with cutting mode of RF. Base was gently curetted and bleeding points were cauterized. Wound was closed after undermining with Prolene™ 5-0 [Figure 2]. Pressure dressing was done which was changed on third day. She was given antibiotic and analgesic for 2 weeks. Stitches removed alternately on 8^th^ and 14^th^ day. Initially for 1 month, patient had mild tenderness and itching which disappeared gradually. After 1-year follow-up patient has had no recurrence.

## DISCUSSION

Various modalities are useful for the treatment of BCC. For the superficial lesions, excision remains treatment of choice. Other modalities can be used in patient where surgery is contraindicated. Nodulo ulcerative type of BCC, also called rodent ulcer, is the common variety and is best managed by simple excision of tumour. Excision by RF, with mild curettage was done in our case, which proved effective and yielded satisfactory therapeutic results.
